# Construction, bioinformatics analysis, and validation of competitive endogenous RNA networks in ulcerative colitis

**DOI:** 10.3389/fgene.2022.951243

**Published:** 2022-08-17

**Authors:** Longcong Dong, Ruibin Zhang, Qin Huang, Yuan Shen, Hongying Li, Shuguang Yu, Qiaofeng Wu

**Affiliations:** ^1^ Acupuncture and Tuina College, Chengdu University of Traditional Chinese Medicine, Chengdu, China; ^2^ Acupuncture and Chronobiology Key Laboratory of Sichuan Province, Chengdu, China

**Keywords:** ulcerative colitis, ceRNA network, WGCNA, bioinformatics, qRT-PCR, immune infiltration

## Abstract

**Background:** Ulcerative colitis (UC) is a common chronic disease of the digestive system. Recently, competitive endogenous RNAs (ceRNAs) have been increasingly used to reveal key mechanisms for the pathogenesis and treatment of UC. However, the role of ceRNA in UC pathogenesis has not been fully clarified. This study aimed to explore the mechanism of the lncRNA-miRNA-mRNA ceRNA network in UC and identify potential biomarkers and therapeutic targets.

**Materials and Methods:** An integrative analysis of mRNA, microRNA (miRNA), and long non-coding RNA (lncRNA) files downloaded from the Gene Expression Omnibus (GEO) was performed. Differentially expressed mRNA (DE-mRNAs), miRNA (DE-miRNAs), and lncRNA (DE-lncRNAs) were investigated between the normal and UC groups by the limma package. A weighted correlation network analysis (WGCNA) was used to identify the relative model for constructing the ceRNA network, and, concurrently, miRWalk and DIANA-LncBase databases were used for target prediction. Consecutively, the Gene Ontology (GO), Kyoto encyclopedia of genes and genomes (KEGG) pathway, and Reactome pathway enrichment analyses, protein-protein interaction (PPI) network, Cytohubba, and ClueGO were performed to identify hub genes. Additionally, we examined the immune infiltration characteristics of UC and the correlation between hub genes and immune cells using the immuCellAI database. Finally, the expression of potential biomarkers of ceRNA was validated via qRT-PCR in an experimental UC model induced by dextran sulfate sodium (DSS).

**Result:** The ceRNA network was constructed by combining four mRNAs, two miRNAs, and two lncRNAs, and the receiver operating characteristic (ROC) analysis showed that two mRNAs (*CTLA4* and *STAT1*) had high diagnostic accuracy (area under the curve [AUC] > 0.9). Furthermore, *CTLA4* up-regulation was positively correlated with the infiltration of immune cells. Finally, as a result of this DSS-induced experimental UC model, *CTLA4*, *MIAT*, and several associate genes expression were consistent with the results of previous bioinformatics analysis, which proved our hypothesis.

**Conclusion:** The investigation of the ceRNA network in this study could provide insight into UC pathogenesis. *CTLA4*, which has immune-related properties, can be a potential biomarker in UC, and *MIAT*/*miR-422a*/*CTLA4* ceRNA networks may play important roles in UC.

## Introduction

Inflammatory bowel disease (IBD), which includes Crohn’s disease (CD) and ulcerative colitis (UC), is a chronic inflammatory disorder of the gastrointestinal (GI) tract characterized by aberrant GI activation (Franzosa et al., 2019; Sun et al., 2019). UC is further characterized by an overactive immune response and destruction of the colorectal epithelium involving many complicated pathological factors (Yu et al., 2021). Pharmacologic therapy can relieve the clinical picture of UC, which mainly comprises abdominal pain, bloody stool, and diarrhea (Kornbluth and Sachar, 2010). Still, modern treatment has certain side effects, and tolerance to treatment may develop among UC patients (Marius et al., 2020; Wang Z et al., 2021). Over the last few decades, UC has become a global disease, with prevalence on every continent rising, increasing a greater economic burden on society (Ng et al., 2017). Unfortunately, the exact pathogenesis of UC remains unclear (Sarvestani et al., 2021).

In recent years, the competing endogenous RNA (ceRNA) hypothesis has proposed the synergistic regulation of different RNAs, including long non-coding RNA (lncRNA), microRNA (miRNA), transcribable pseudogenes, and circular RNAs (Salmena et al., 2011; Thomson and Dinger, 2016). There is increasing evidence that lncRNAs play critical roles in gene regulation, cell biology, human development, and several diseases (Mercer et al., 2022). LncRNAs, miRNA molecular “sponges,” can indirectly modulate gene expression through their ability to compete and sequester miRNAs, thereby inhibiting the miRNA-mediated suppression of target mRNAs (Salmena et al., 2011). In the current research, ceRNA has been closely related to immune diseases, such as rheumatoid arthritis (RA), Sjögren’s syndrome (SS), systemic lupus erythematosus (SLE), and systemic sclerosis (SSc) (Chen et al., 2021; Song et al., 2021; Wu et al., 2021; Zhang Y et al., 2020). Similarly, ceRNA has also been recently identified as one of the key mechanisms involved in the development of IBD (Nie and Zhao, 2020; Liu et al., 2021).

In the present study, we obtained the original data from the NCBI Gene Expression Omnibus (GEO) DataSets database, including the UC group and the normal control group. Then, we obtained differentially expressed (DE)-lncRNAs, DE-miRNAs, and DE-mRNAs through the gene differential expression analysis. We aimed to construct and explore a complete lncRNA-miRNA-mRNA network to determine the specific immune infiltration characteristics of UC. Finally, we validated the bioinformatics analysis results using the UC model induced by dextran sulfate sodium (DSS) to provide a basis for further understanding of UC molecular mechanism and identifying potential biomarkers.

## Materials and methods

### GEO dataset selection

**FIGURE 1:**
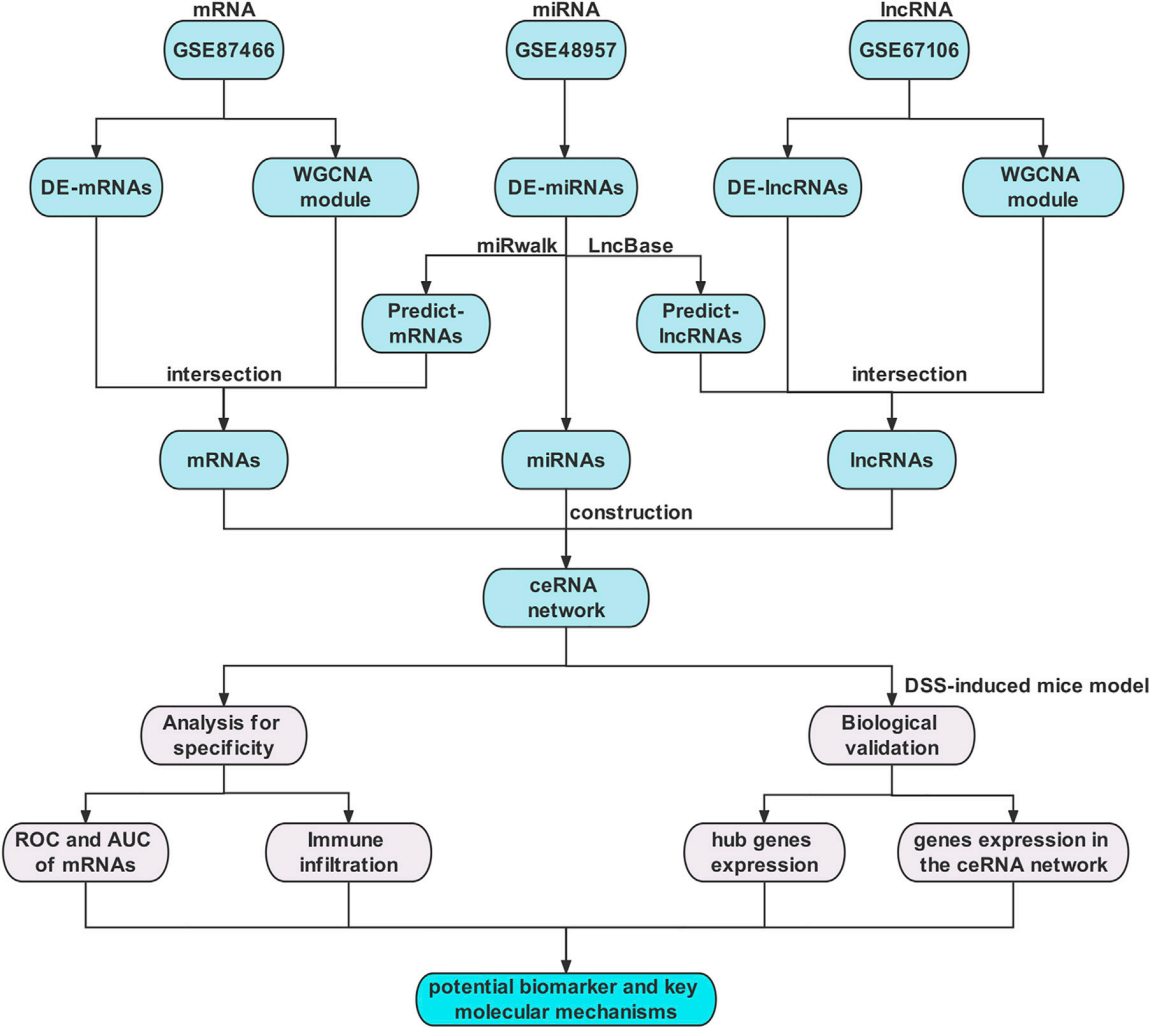
Flow chart of the overall analysis.

A flowchart of the study design is depicted in Figure 1. The datasets of mRNAs, miRNAs, and lncRNAs were downloaded from the GEO database (http://www.ncbi.nlm.nih.gov/geo). For a better understanding of the ceRNA mechanism in UC, the independent datasets of GSE87466 for mRNAs, GSE48957 for miRNAs, and GSE67106 for lncRNAs were selected, including 87 UC tissues and 21 normal tissues, 10 UC tissues and 10 normal tissues, and 21 UC tissues and 22 normal tissues from intestinal biopsies, respectively. Table 1 lists the basic details of these three datasets.

**TABLE 1:** Basic information of three UC microarray datasets.

Datasets	RNA type	Platform	Experiment type	Sample size	Sample source	Organism	Year
GSE48957	miRNA	GPL14613	Non-coding RNA profiling by array	10 UC/10 normal	Colonic mucosal	Homo sapiens	2015
GSE87466	mRNA	GPL13158	Expression profiling by array	87 UC/21 normal	Colonic mucosal	Homo sapiens	2018
GSE67106	lncRNA	GPL19920	Non-coding RNA profiling by array	21 UC/22 normal	Colonic mucosal	Homo sapiens	2015

### Differential expression analysis and weighted correlation network analysis (WGCNA)

The limma package was used for differential expression analysis with a *p*-value of <0.05 and |log2 (fold change) | > 1 to define DE-mRNAs, DE-miRNAs, and DE-lncRNAs (Davis and Meltzer, 2007). The relative importance of mRNAs, lncRNAs, and their module membership was assessed using the WGCNA package (Langfelder and Horvath, 2008). The minimum number of genes per module was set to 30. Clustering by hierarchical analysis identified gene co-expression modules, which were then colored arbitrarily for identification.

### Functional enrichment analysis and protein-protein interaction (PPI) network construction

DAVID (http://david.abcc.ncifcrf.gov) was used to analyze the Kyoto encyclopedia of genes and genomes (KEGG) and Gene Ontology (GO) terms (Yu et al., 2012). A high-confidence set of human PPI networks was constructed by the STRING database (https://string-db.org, version 11.5) (Szklarczyk et al., 2021). A CytoHubba plug-in (http://apps.cytoscape.org/apps/cytohubba) in Cytoscape (version 3.9.1) was applied to identify the top 10 hub genes in PPI networks through the degree algorithm (Chin et al., 2014; Otasek et al., 2019). In ClueGo (http://apps.cytoscape.org/apps/cluego), kappa statistics were used to construct and compare networks of functionally related GO terms and Reactome pathways (Bindea et al., 2009; Croft et al., 2014; Szklarczyk et al., 2021).

### ceRNA network construction

We used the miRWalk database (http://mirwalk.umm.uni-heidelberg.de) to predict the interaction between miRNA and mRNA (Dweep and Gretz, 2015). We predicted lncRNA-miRNA interactions via the DIANA-LncBase v2 database (http://www.microrna.gr/LncBase) (Paraskevopoulou et al., 2016). DE-mRNA, DE-lncRNA, DE-miRNA, WGCNA module, predict lncRNA, and predict mRNA were selected to construct the ceRNA network. A visualization of the ceRNA network was performed by Cytoscape (Otasek et al., 2019).

### Immune infiltration analysis

The immuCellAI database (http://bioinfo.life.hust.edu.cn/web/ImmuCellAI) was used to estimate differential immune cell infiltration between the normal and UC groups (Miao et al., 2020).

### DSS-induced UC mice model

Male C57BL/6J mice (26 ± 2 g) were obtained from GemPharmatech Co., Ltd. (Chengdu, China). The animals were fed standard rodent chow and water *ad libitum* under ambient temperature (23 ± 1 °C) and a 12-h light/dark cycle in their pathogen-free enclosure. The mice were randomized into the normal and UC groups. In the UC group, mice were administered 2.5% DSS (MP Biomedicals, CA, United States ) for 7 days, while the normal group only received distilled water. Daily measurements of food intake, body weight, stool consistency, and fecal bleeding were taken.

### Disease activity index (DAI)

DAI was calculated by combining body weight loss, feces status, and occult levels. The scores for each subscale are shown in Supplementary Table S1.

### Enzyme-linked immunosorbent assay (ELISA)

Centrifugation at 3,000 rpm for 20 min was used to collect supernatants from the different groups. In accordance with the manufacturer’s protocols, ELISA kits (Elabscience, Wuhan, China) were used to detect tumor necrosis factor (TNF)-α and interferon (IFN)-γ levels in the supernatant. Then, the absorption coefficients were applied to calculate TNF-α and IFN-γ concentrations.

### Hematoxylin and eosin (HE) staining

In the normal and UC groups, the colon tissues were fixed in buffered 4% formalin for 2 days. The samples were sectioned at a thickness of 5 mm and stained with HE for histopathological examination using a light microscope (Olympus Corporation, Tokyo, Japan). Supplementary Table S2 presents the scores for each subscale.

### Real-time quantitative PCR (qRT-PCR)

MolPure^®^ TRIeasyTM Plus Total RNA Kit (YEASEN, Shanghai, China) was used to isolate total mRNA from colon tissues, and cDNA was prepared using Hifair®III first Strand cDNA Synthesis SuperMix for qPCR (YEASEN, Shanghai, China) according to the manufacturer’s instructions. We then analyzed the resulting cDNA by RT-qPCR using Hieff UNICON universal Blue qPCR SYBR Green Master Mix (YEASEN, Shanghai, China). The primer sequences are seen in Supplementary Table S3. Target mRNA expression levels were analyzed using the comparative 2-Ct method by normalizing to levels of *β-actin*.

### Western blot (WB)

Western blot analysis was performed as previously described (Sun et al., 2017). The protein extracts of mice colon were prepared using a lysis buffer supplemented with ethylenediaminetetraacetic acid (EDTA)-free complete protease inhibitors. We extracted proteins and subjected them to 10% sodium dodecyl sulfate-polyacrylamide gels (SDS-PAGE). Then, a polyvinylidene difluoride membrane was used to transfer the bands. After blocking with 5% nonfat dry milk in Tris-buffered saline supplemented with 0.1% Tween 20 for 2 h at room temperature, the membranes were incubated overnight at 4 °C with the anti-CTLA4 antibody (ab237712, 1:1000 Abcam, MA, United States ). The membranes were then incubated with a secondary antibody (HS101-01, 1:5000, TRAN, Beijing, China) at 37 °C for 2 h. Western blotting of the same membranes with an anti-β-actin antibody (20536-1-AP, 1:1000, Proteintech, Wuhan, China) was used to normalize the results.

### Immunofluorescence (IF)

Sections of samples were deparaffinized in xylene and rehydrated in alcohol series with decreasing concentrations. We washed sections three times in PBS for 5 min, then blotted them with 5% goat serum for 1 h at 37°C. Slices were incubated with the Anti-CTLA4 antibody (ab237712, 1:200, Abcam, MA, United States ) overnight at 4 °C. These sections were washed three times in phosphate-buffered saline (PBS) and then incubated with Cy3–conjugated Affinipure Goat Anti-Rabbit IgG (SA00009-2, 1:100, Proteintech, Wuhan, China) at 37°C for 2 h. Then, the sections were rinsed three more times and stained for 5 minutes with DAPI (Biyuntian, Shanghai, China). The results were imaged by Pannoramic 250FLASH (3DHISTECH, Budapest, Hungary).

### Statistical analysis

Statistical analysis was performed using SPSS software (version 24.0, SPSS Inc., Chicago, United States ), and pictures were drawn using GraphPad Prism software (version 8.0, GraphPad Prism Software Inc., CA, United States ). PPI network construction and analysis were performed according to the required confidence threshold set (combined score) of >0.9. Spearman correlation analysis was used to analyze the correlation between hub genes and immune cells. The receiver operating characteristic (ROC) curves were generated with the R package pROC, and the corresponding area under the curve (AUC) values were calculated. The negatively related mRNA-lncRNA co-expression interactions and positively related mRNA-miRNA and lncRNA-miRNA were removed to construct the lncRNA-miRNA-mRNA ceRNA network construction. The qRT-PCR and WB results were analyzed by Student’s t tests. All performed statistical tests were two-sided, and a *P* of >0.05 was chosen as the threshold of statistical significance. The results were presented as mean ± standard deviation (SD).

## Results

### Identification of DE-miRNAs

**FIGURE 2:**
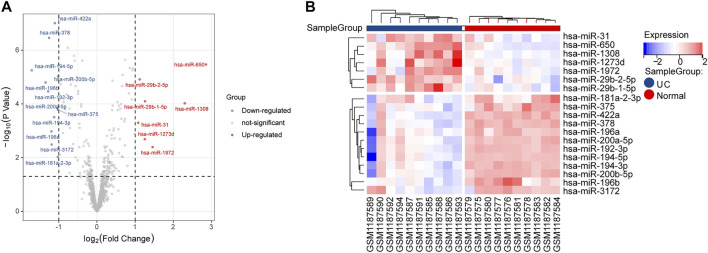
DE-miRNA screening. **(A)** Volcano plots of DE-miRNAs from GSE48957. **(B)** Heatmap of DE-miRNAs from GSE48957.

In this study, the expression levels of GSE48957 miRNAs, which were obtained from the GEO database, were explored. As shown in the volcano plot and the heatmap, we found that 7 DE-miRNAs were significantly increased, and 12 DE-miRNAs were decreased in the UC group compared with the normal group (Figure 2 and Table 2).

**TABLE 2:** Difference analysis results of miRNAs in dataset GSE48957.

Gene symbol	Log_2_FC	*p*-value	Change
*hsa-miR-650*	2.8575	3.40E-06	up
*hsa-miR-1308*	2.2995	9.48E-05	up
*hsa-miR-1972*	1.458	0.004053315	up
*hsa-miR-29b-1-5p*	1.26	7.95E-05	up
*hsa-miR-1273d*	1.254	0.002056625	up
*hsa-miR-29b-2-5p*	1.119	1.22E-05	up
*hsa-miR-31*	1.080333333	0.00050246	up
*hsa-miR-200b-5p*	−1.01825	9.48E-06	down
*hsa-miR-375*	−1.022	0.000164088	down
*hsa-miR-181a-2-3p*	−1.024	0.009395976	down
*hsa-miR-200a-5p*	−1.039333333	0.000142113	down
*hsa-miR-422a*	−1.104	9.86E-08	down
*hsa-miR-194-3p*	−1.12	0.000310773	down
*hsa-miR-3172*	−1.187	0.003248514	down
*hsa-miR-196a*	−1.193	0.001050684	down
*hsa-miR-378*	−1.254	3.49E-07	down
*hsa-miR-192-3p*	−1.278333333	3.42E-05	down
*hsa-miR-196b*	−1.346	1.59E-05	down
*hsa-miR-194-5p*	−1.709	5.61E-06	down

### Enrichment analysis of DE-mRNAs

**FIGURE 3:**
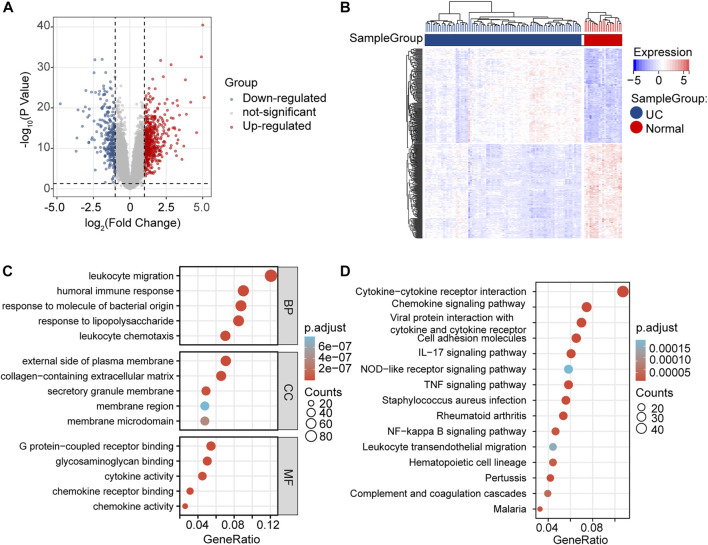
DE-mRNAs screening and enrichment analysis. **(A)** Volcano plots of DE-mRNAs from GSE87466. **(B)** Heatmap of DE-mRNAs from GSE87466. **(C)** BP, CC, and MF presented the top five significant terms of GO enrichment analysis. **(D)** Top 15 significant terms of KEGG enrichment analysis.

We investigated the expression levels of mRNAs related to GSE87466, which were obtained from the GEO database. As shown in the volcano plot and the heatmap, we found that 537 DE-mRNAs were significantly increased, and 270 DE-mRNAs were decreased in the UC group compared with the normal group (Figures 3A, B). Then, a functional enrichment analysis of GO and KEGG was performed to explore the potential function of 807 DE-mRNAs. Among them, the top five BP, top 5 MF, and top 5 CC GO terms are shown in Figure 3C. “Leukocyte migration,” “humoral immune response,” “response to molecule of bacterial origin,” “leukocyte chemotaxis,” and “response to lipopolysaccharide” were the top five enriched terms of BP. The top five GO terms on CC were “external side of plasma membrane,” “collagen-containing extracellular matrix,” “secretory granule membrane,” “membrane microdomain,” and “membrane region.” In categories of MF, “chemokine receptor binding,” “chemokine activity,” “glycosaminoglycan binding,” “G protein-coupled receptor binding,” and “cytokine activity” were major terms. KEGG analysis showed that obvious DE-mRNAs were significantly enriched in 37 KEGG pathways, such as “viral protein interaction with cytokine and cytokine receptor,” “IL-17 signaling pathway,” and “cytokine-cytokine receptor interaction,” as shown in Figure 3D.

### Identification of mRNAs co-expression modules via WGCNA

**FIGURE 4:**
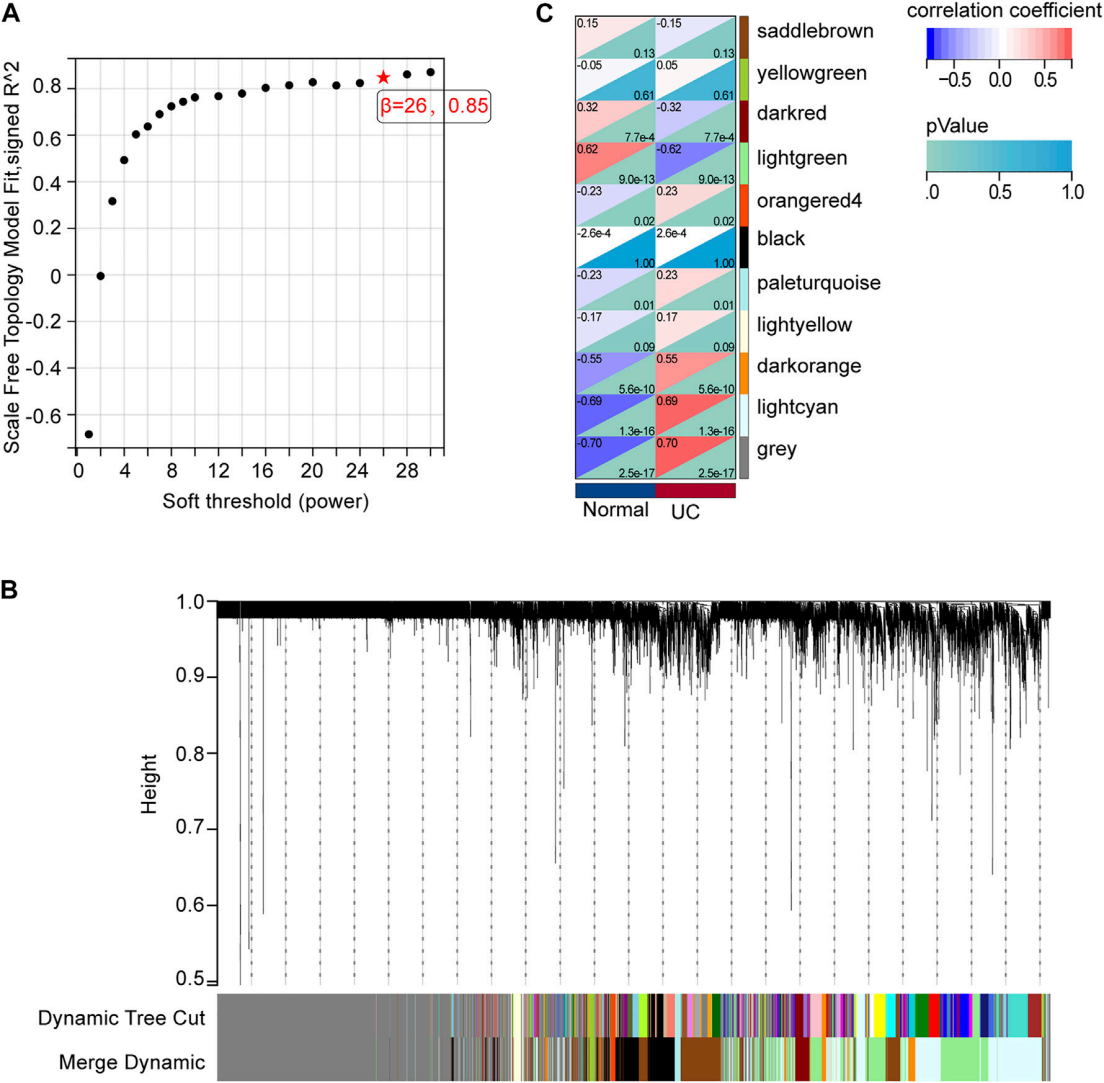
mRNA modules analyzed by WGCNA. **(A)** Determination of soft-thresholding power. **(B)** Clustering dendrogram. **(C)** The module-trait relationships.

We analyzed the gene co-expression networks and identified gene modules using WGCNA to further confirm the key mRNAs in DE-mRNAs. When *β* was 26 (a soft threshold), the scale-free R2 was 0.85 to obtain a higher average connectivity degree (Figure 4A). Ten different gene co-expression modules were determined in DE-mRNAs after the insignificant gray module was excluded (Figure 4B). As shown in Figure 4C, lightcyan modules showed the most positive correlation (module trait correlation = 0.69), and lightgreen modules exhibited a negative correlation (module trait correlation = −0.62) with the UC group.

### Enrichment analysis of interacted DE-mRNAs and screened hub DE-mRNAs

**FIGURE 5:**
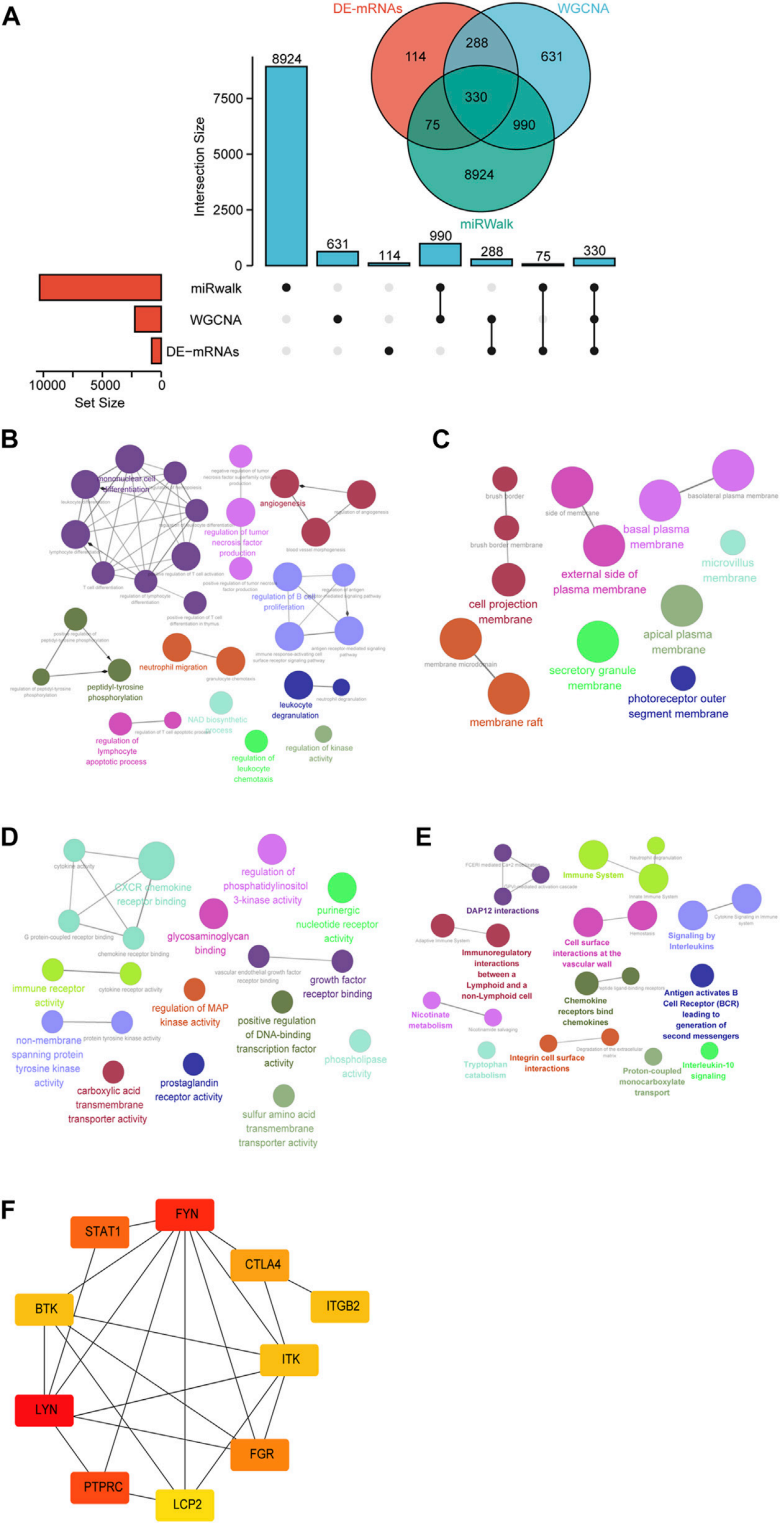
Identification of candidate overlapping mRNA and function annotation. **(A)** The intersection of DE-mRNAs, WGCNA modules, and miRwalk-predict. **(B)** BP of overlapping mRNA visualized by ClueGO. **(C)** CC of overlapping mRNA visualized by ClueGO. **(D)** MF of overlapping mRNA visualized by ClueGO. **(E)** Reactome pathway of overlapping mRNA visualized by ClueGO. **(F)** Hub genes were identified in the overlapping mRNA via cytohubba.

To further define the DE-mRNAs of UC, the mRNAs targeted by DE-miRNAs were predicted by using the miRwalk database, and 330 DE-mRNAs were defined in the intersection between DE-mRNAs, WGCNA modules, and DE-miRNA-predict-mRNA (Figure 5A). To further understand the biological functions of these mRNAs, GO functional enrichment and Reactome pathway enrichment were conducted by ClueGO of Cytoscape. As can be seen in Figure 5B and Supplementary Figure S1A, within the BP category, “mononuclear cell differentiation” (29.03%) was the most dominant group, followed by “regulation of B cell proliferation” (12.9%), “peptidyl-tyrosine phosphorylation” (9.68%), “angiogenesis” (9.68%), and “regulation of tumor necrosis factor production” (9.68%). The top CC categories were “cell projection membrane” (23.08%), “basal plasma membrane” (15.38%), “membrane raft” (15.38%), and “external side of plasma membrane” (15.38%), as indicated in Figure 5C and Supplementary Figure S1B. MF mainly involved “CXCR chemokine receptor binding” (21.05%), “non-membrane spanning protein tyrosine kinase activity” (10.53%), “immune receptor activity” (10.53%), and “growth factor receptor binding” (10.53%) (Figure 5D and Supplementary Figure S1C). Meanwhile, we found that “immune system” (13.64%), “DAP12 interactions” (13.64%), “signaling by interleukins” (9.09%), and “chemokine receptors bind chemokines” (9.09%) were significantly enriched in Reactome pathway terms, as shown in Figure 5E and Supplementary Figure S1D.

To further investigate the functions of these mRNAs, we performed a PPI network analysis on the String database and selected mRNAs with the highest confidence. A total of 103 DE-mRNAs were filtered into the DE-mRNAs’ PPI network complex containing 103 nodes and 142 edges (Supplementary Figure S2). Subsequently, CytoHubba was used to identify the hub mRNAs with the highest connectivity in the PPI network. The top 10 hub mRNAs, such as *LYN*, *FYN*, *PTPRC*, *STAT1*, *FGR*, *CTLA4*, *BTK*, *ITK*, *ITGB2*, and *LCP2,* are shown in Figure 5F.

### Identifying interacted lncRNAs via DE-lncRNAs, WGCNA, and predicted lncRNAs

**FIGURE 6:**
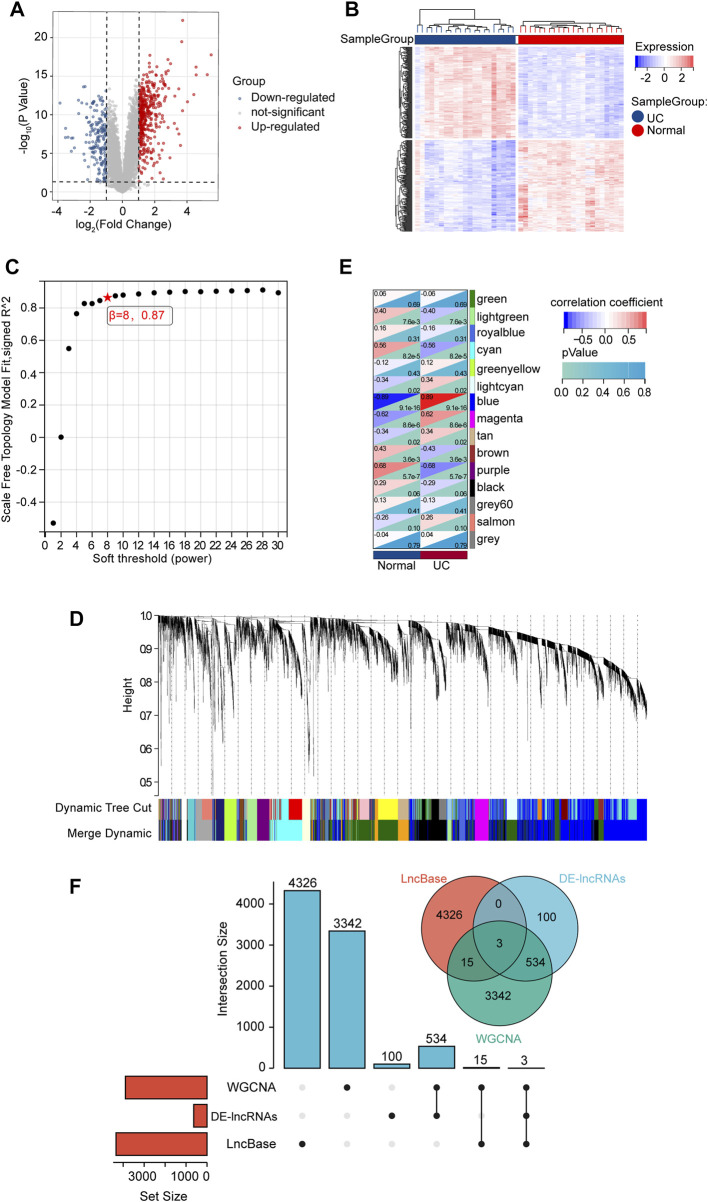
DE-lncRNA screening and identification of candidate overlapping DE-lncRNA. **(A)** Volcano plots of DE-mRNAs from GSE67106. **(B)** Heatmap of DE-mRNAs from GSE67106. **(C)** Determination of soft-thresholding power. **(D)** Clustering dendrogram. **(E)** The module-trait relationships. **(F)** The intersection of DE-lncRNAs, WGCNA modules, and LncBase-predict.

To search for the lncRNAs involved in our study, we analyzed the expression of GSE67106 lncRNAs from the GEO dataset. 637 DE-lncRNAs were obtained, with 441 up-regulated and 196 down-regulated DE-lncRNAs (Figures 6A, B). In WGCNA analysis, the soft threshold power was selected as eight to ensure a scale-free network distribution (scale-free index R2 = 0.87) (Figure 6C). The lncRNAs co-expression modules and the corresponding hierarchical clustering dendrograms of these lncRNAs are shown in Figure 6D. As shown in Figure 6E, blue modules showed the most positive correlation (module trait correlation = 0.89), and purple modules exhibited a negative correlation (module trait correlation = −0.68) with the UC group. The DE-lncRNAs interacted with WGCNA modules, and predicted lncRNAs, as shown in Figure 6F, were used to construct the ceRNA network in the next step.

### CeRNA network constructed

**FIGURE 7:**
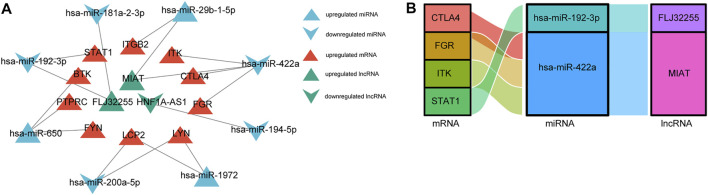
The construction of **(A)** ceRNA network. The triangle represents up-regulate, and v represents down-regulate. Red color represents mRNAs, blue color represents miRNAs, and green color represents lncRNAs. **(B)** Sankey diagram of final ceRNA network. The squareness represents lncRNAs, miRNAs, and mRNAs, and the size indicates their degree of connection.

The above-described interactions between lncRNA-miRNA, miRNA-mRNA, and lncRNA-mRNA were used to establish a lncRNA-miRNA-mRNA network via Cytoscape. We constructed a ceRNA network based on eight miRNA nodes, 10 mRNA nodes, and three lncRNA nodes, as shown in Figure 7A. We finally identified the ceRNA network among four mRNAs, two miRNAs, and two lncRNAs (Figure 7B).

### ROC analysis and validation

**FIGURE 8:**
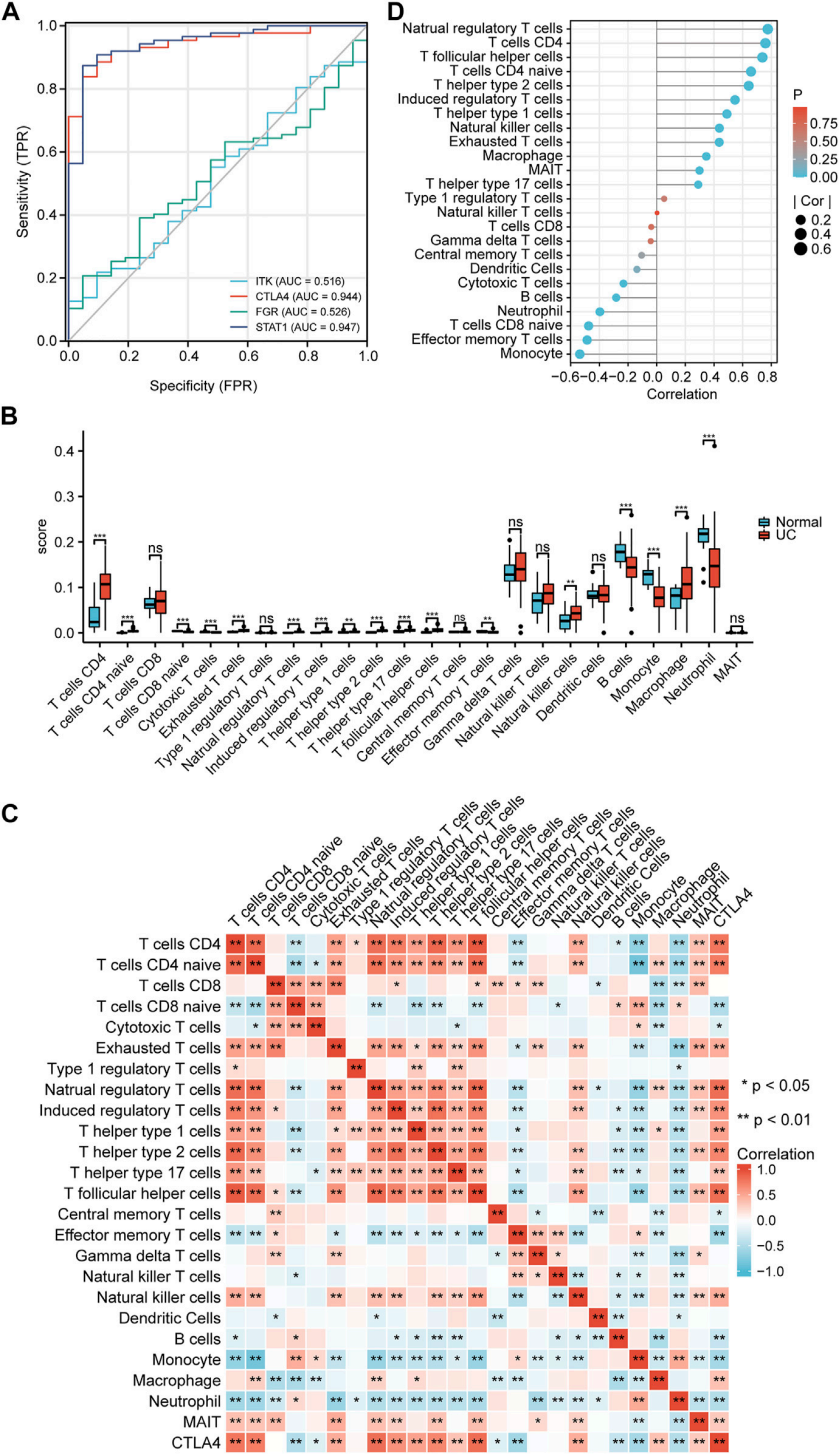
Validation and immune infiltration analysis. **(A)** ROC of mRNAs in the ceRNA network. **(B)** The differential of immune infiltration between the UC and normal groups. **(C)** Correlation matrix of immune cell proportions and *CTLA4*. **(D)** Lollipop chart of the correlation between immune cells and *CTLA4*. ^*^ *p* < 0.05, ^**^ *p* < 0.01.

Representative ROC curves for the finally identified ceRNA network mRNAs (*ITK*, *CTLA4*, *FGR*, and *STAT1*) are shown in Figure 8A. We found that except for *ITK* and *FGR*, the AUCs for the responsiveness of *CTLA4* and *STAT1* were >0.90, which demonstrated excellent responsiveness to predict UC.

### Immune infiltration analysis

To further test our hypothesis, the immune cell abundance between the normal and UC groups was calculated using the immuCellAI database. As shown in Figure 8B, the fraction of CD4^+^ T cells, CD4 naïve T cells, exhausted T cells, natural regulatory T cells, induced regulatory T cells, T helper type 1 cells, T helper type 2 cells, T helper type 17 cells, T follicular helper cells, natural killer cells, and macrophages in the UC group were higher than in the normal group while that of CD8 naïve T cells, cytotoxic T cells, effector memory T cells, monocytes, and neutrophils was lower. *CTLA4* expression levels correlated well with natural regulatory T follicular helper cells, CD4^+^ T cells, natural regulatory T cells, CD4 naïve T cells, T helper type 2 cells, induced regulatory T cells, and monocytes (Pearsons correlation coefficient >0.5, *p* < 0.01) (Figures 8C, D).

### DSS-induced mice model validation

**FIGURE 9:**
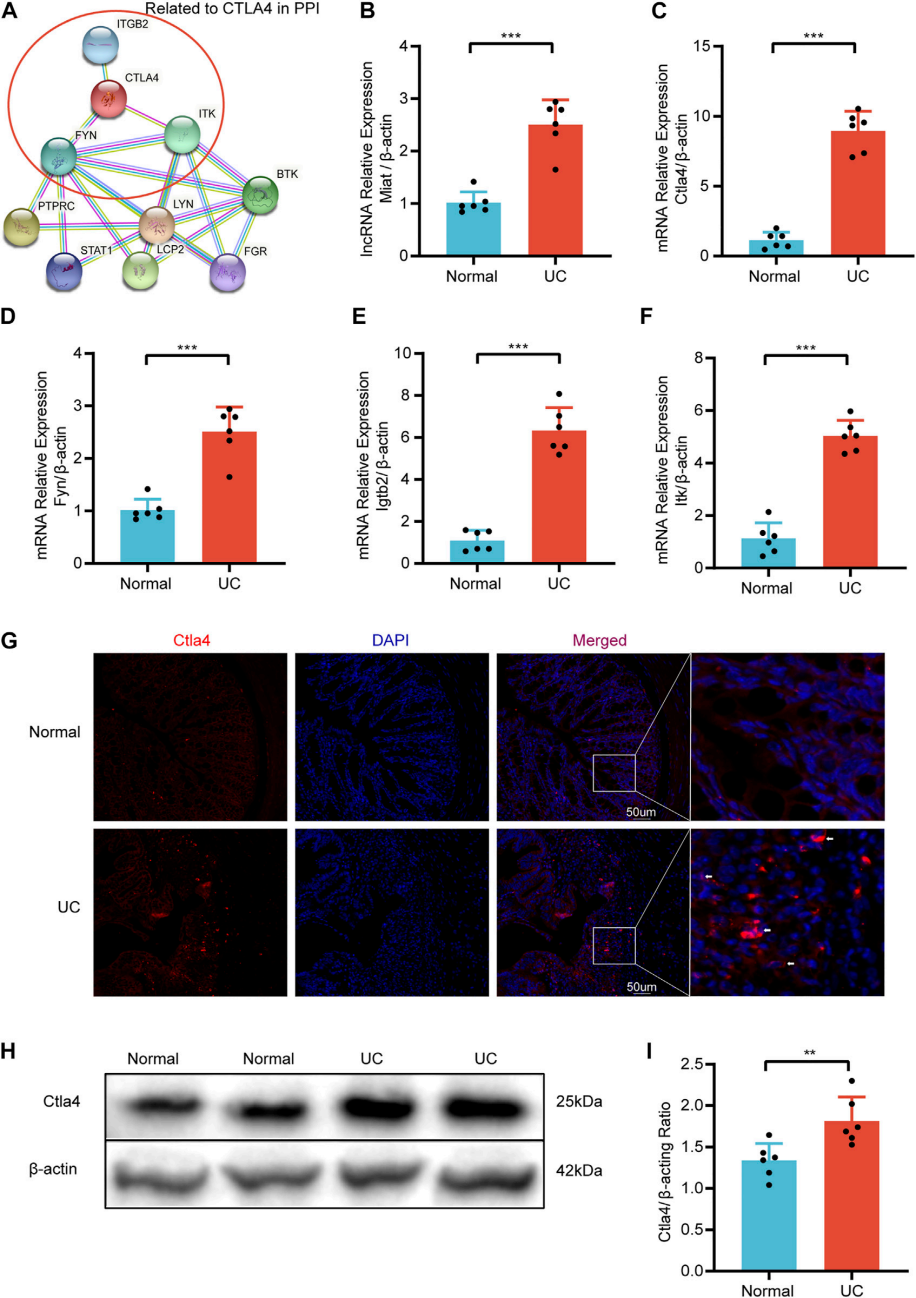
The relative expression was validated via qRT-PCR. **(A)** Hub mRNAs Related to *CTLA4* in PPI. The relative expression of *Miat* **(B)**, *Clta4* **(C)**, *Fyn* **(D)**, *Igtb2* **(E)**, and *Itk* **(F)** in DSS-induced mice and Normal mice. **(G)** Representative immunofluorescence image of *CTLA4* between DSS-induced mice and Normal mice. Red, *Ctla4*; blue, DAPI nuclear staining (n = 6/group). **(H)** Representative western blot image of *Ctla4* between DSS-induced mice and Normal mice. **(I)** Quantification of western blot analysis. Dates are shown as mean ± SD. ^**^ *p* < 0.01, ^***^ *p* < 0.001.

After the DSS-induced mice model establishment (Supplementary Figure S3), we examined the mRNA expression levels of *CTLA4* and three strongly correlated mRNAs (*FYN*, *ITK*, and *IGTB2*) in the top 10 hub mRNAs (Figure 9A) and the lncRNA expression levels of myocardial infarction-associated transcript (*MIAT*) with qRT-PCR to verify the dataset’ reliability. Compared with the normal group, the expression levels of *CTLA4*, *FYN*, *ITK*, *ITGB2,* and *MIAT* in the UC group were significantly increased (Figures 9B–F). Additionally, IF and WB confirmed that the critical hub gene *CTLA4* was significantly elevated at the protein level compared to the normal group (Figures 9G–I).

## Discussion

UC is a chronic inflammatory disease characterized by dysregulated immune responses in the colon with a rising incidence worldwide (Ungaro et al., 2017). Due to the complex pathogenesis and unclear pathophysiological mechanism of UC, the current clinical treatment has mainly focused on alleviating the symptoms (Sarvestani et al., 2021; Ungaro et al., 2017). CeRNA cross-talk, mediated by miRNA in complex ceRNA networks, and the interactions between miRNA and lncRNA have been shown to play critical roles in the pathogenesis of various immune disorders (Chen et al., 2021; Salmena et al., 2011; Song et al., 2021; Thomson and Dinger, 2016; Wu et al., 2021; Zhang Y et al., 2020). Studies have shown that lncRNA-*ITSN1-2* acts as a competing endogenous RNA for *IL-23R* via sponging *miR-125a*, and lncRNA *meg3* serves as a competing RNA for *IL-10* via sponging *miR-98-5p* in IBD (Nie and Zhao, 2020; Wang Y et al., 2021). In UC-associated colon cancer, *CXCR4* promotes the progression of the disease by recruiting immune cells and enhancing cytoskeletal remodeling through the lncRNA *XIST*/*miR-133a-3p*/*RhoA* signaling (Yu et al., 2019). Therefore, the construction of the ceRNA is essential to understanding the immune-regulation mechanisms and exploring potential biomarkers in UC.

In the present study, DE-mRNAs, DE-miRNAs, and DE-lncRNAs were identified in UC and normal colon tissues from GSE87466, GSE48957, and GSE67106, respectively. Next, UC-related modules were selected by WGCNA. Additionally, we used DE-miRNAs to predict interacted mRNAs and lncRNAs through the miRwalk and LncBase databases. Intersection-mRNAs were identified at the intersection of DE-mRNAs, mRNAs in the WGCNA module, and predicted mRNAs. Similarly, intersection-lncRNAs were also obtained by the above-mentioned method. Thus, we proposed a regulatory network of ceRNA relying on intersection-mRNAs, intersection-lncRNAs, and DE-miRNAs. Finally, according to the competition rules of ceRNA, we further established a ceRNA regulatory network including four mRNAs, two lncRNAs, and two miRNAs. GSE87466 from colon tissues was validated by the ROC analysis to determine whether eight mRNAs from ceRNA were used as UC biomarkers. The results showed that AUCs of *CTLA4* and *STAT1* exceeded 0.9, suggesting that these genes could be potential biomarkers of UC.

CTLA4, predominantly found in intracellular vesicles in FoxP3^+^ T regulatory cells or activated conventional T cells, is a key regulator of autoimmunity and acts as an immune checkpoint (Hodi et al., 2014; Rowshanravan et al., 2018; Wang et al., 2015). With the wide application of immune checkpoint inhibitors (ICIs) in treating various diseases, colitis, as one of the side effects of ICIs, has also received more and more attention (Ouyang et al., 2021). For example, an observational study focusing on gastrointestinal immune-related adverse events of ICIs has suggested that patients treated with anti-CTLA4 antibodies show a higher incidence of events and more severe symptoms (Yamada et al., 2021). Furthermore, compared with non-inflamed individuals, *CTLA4* is increased in biopsies from UC (Lushnikova et al., 2021). In fact, as shown by the results of immune infiltration analysis, the expression level of *CTLA4* is strongly associated with many types of immune cells in UC. Studies have confirmed that *CTLA4* may affect the progression of UC by secreting IL-10 from regulatory T cells (Coquerelle et al., 2009; Liu et al., 2003). Moreover, *CTLA4* can elicit a CD4^+^ T cell response, blocking T cell homing and increasing UC severity (Zhang S et al., 2020). *CTLA-4* has a particularly important role in the microbe-rich barriers of the intestine (Rowshanravan et al., 2018). Patients treated with anti-CTLA-4 have shown fluctuating levels of antibodies against gastrointestinal commensals, suggesting dysregulated mucosal immunity with *CTLA-4* blockade (Berman et al., 2010). These results suggest that *CTLA4* is likely to be an immune-related biomarker candidate for UC.

In this study, we confirmed that *CTLA4* expression was regulated via the *MIAT*/*miR-422a*/*CTLA4* ceRNA network. In the previous study, lncRNA *MIAT*, as a genetic risk factor for myocardial infarction, has become the focus of extensive studies (Yang et al., 2021). With the deepening of research, it has been found that *MIAT* is also closely related to the occurrence and development of inflammation (Meydan et al., 2018; Qu et al., 2021). Interestingly, *MIAT* has been positively correlated with the number of immune cells in various diseases, such as B cells, T lymphocytes, and macrophages, and the expression of *CTLA4* in the ceRNA network constructed in this study (Peng et al., 2020; Ye et al., 2021). Furthermore, *MIAT* was up-regulated in a time-dependent manner during the differentiation of bone marrow mesenchymal stem cells into endothelial cells (Wang et al., 2018). *MIAT* may also function through a feedback loop with *AKT* to regulate human lens epithelial cell function by increasing oxidative stress and inflammation factors (Shen et al., 2016). For those reasons, we considered that the role of *MIAT* in UC is not only in regulating inflammatory immunity but also in regulating the function of intestinal epithelial cells and vascular endothelial cells. *MIAT* acts as a post-transcriptional regulator by interacting with *miR-422a*, which has been reported as a kind of important molecule to participate in the formation of the immune landscape (Peng et al., 2020). Research has indicated that *miR-422a* may affect the inflammatory response in UC colon tissue by regulating the expression level of *IL-33*, which has been shown to cause a T helper 2-like cytokine response in immune cells (Dubois-Camacho et al., 2019; Kobori et al., 2010). Long-standing IBD has an approximately 2–3-fold increased risk of colorectal cancer (CRC) (Shah and Itzkowitz, 2022). Interestingly, it has been found that *miR-422a* also regulates the expression of *AKT1*, *MAPK1*, *MAPKK6*, and other inflammatory-related factors in CRC, thereby affecting the proliferation and apoptosis of CRC cells (Li et al., 2018; Liang et al., 2021; Wei et al., 2017). Additionally, studies on the expression profile of miRNAs, which were not only in CRC tissue but also in the serum, have suggested that *miR-422a* is highly dysregulated in CRC patients compared to healthy individuals (Faltejskova et al., 2012; Qin et al., 2015; Zheng et al., 2014). Therefore, we believe that *miR-422a* plays a vital role in both UC and CRC and is an essential biomarker for the prognosis of colitis.

The DSS-induced UC model is widely used due to its simplicity, reliability, and many similarities to human UC (Chassaing et al., 2014). Therefore, we established DSS-induced UC in mice and validated the expression of *Miat*, *Ctla4*, and associated mRNAs, such as *Fyn*, *Itk* and *Itgb2*, in this model. The results were consistent with the previous finding and revealed that the expression level of the RNA expression levels of *Miat*, *Ctla4*, *Fyn*, *Itk*, and *Itgb2* were obviously increased in the UC model by qRT-PCR. Moreover, IF and WB verified that the changing trend of *CTLA4* was consistent with mRNA at the protein level, which further confirmed our hypothesis. Thus, this study shows that the *MIAT*/miR-*422a*/*CTLA4* ceRNA network may play an essential role in the immune responses in UC.

## Conclusion

In summary, we constructed a *MIAT*/*miR-422a*/*CTLA4* ceRNA network in UC. We found an exact relationship between *CTLA4* and *MIAT*, which is a novel insight into the molecular mechanism of UC. *CTLA4* was significantly positively correlated with up-regulated immune cells, and *MIAT* was lncRNA closely related to vascular lesions. Our results help improve understanding of the UC mechanism and provide future potential biomarkers for UC diagnosis and prognosis.

## Data Availability

The datasets presented in this study can be found in online repositories. The names of the repository/repositories and accession number(s) can be found below: Gene Expression Omnibus, accession number GSE87466, GSE48957, and GSE67106.
