# Supplementary material for: Construction, bioinformatics analysis, and validation of competitive endogenous RNA networks in ulcerative colitis
Source: Front Genet. 2022 Aug 17;13:951243. doi: 10.3389/fgene.2022.951243 (PMC9428148; doi:10.3389/fgene.2022.951243)
Supplement: Supplementary file 1 [file Table1.DOCX]

Supplementary Table S1. Scores of disease activity index (DAI) of colitis

| Feature | Score | Description |
| --- | --- | --- |
| Body weight loss | 0 | 0% |
|  | 1 | 1–5% |
|  | 2 | 6–10% |
|  | 3 | 11–15% |
|  | 4 | >15% |
| Feces status | 0 | Normal |
|  | 2 | Loose stools |
|  | 4 | Watery stool |
| Occult/Bloody stools | 0 | Normal |
|  | 2 | Hemoccult positive |
|  | 4 | Hematochezia with naked eyes |
