# Supplementary figures and images for: Construction, bioinformatics analysis, and validation of competitive endogenous RNA networks in ulcerative colitis
Source: Front Genet. 2022 Aug 17;13:951243. doi: 10.3389/fgene.2022.951243 (PMC9428148; doi:10.3389/fgene.2022.951243)

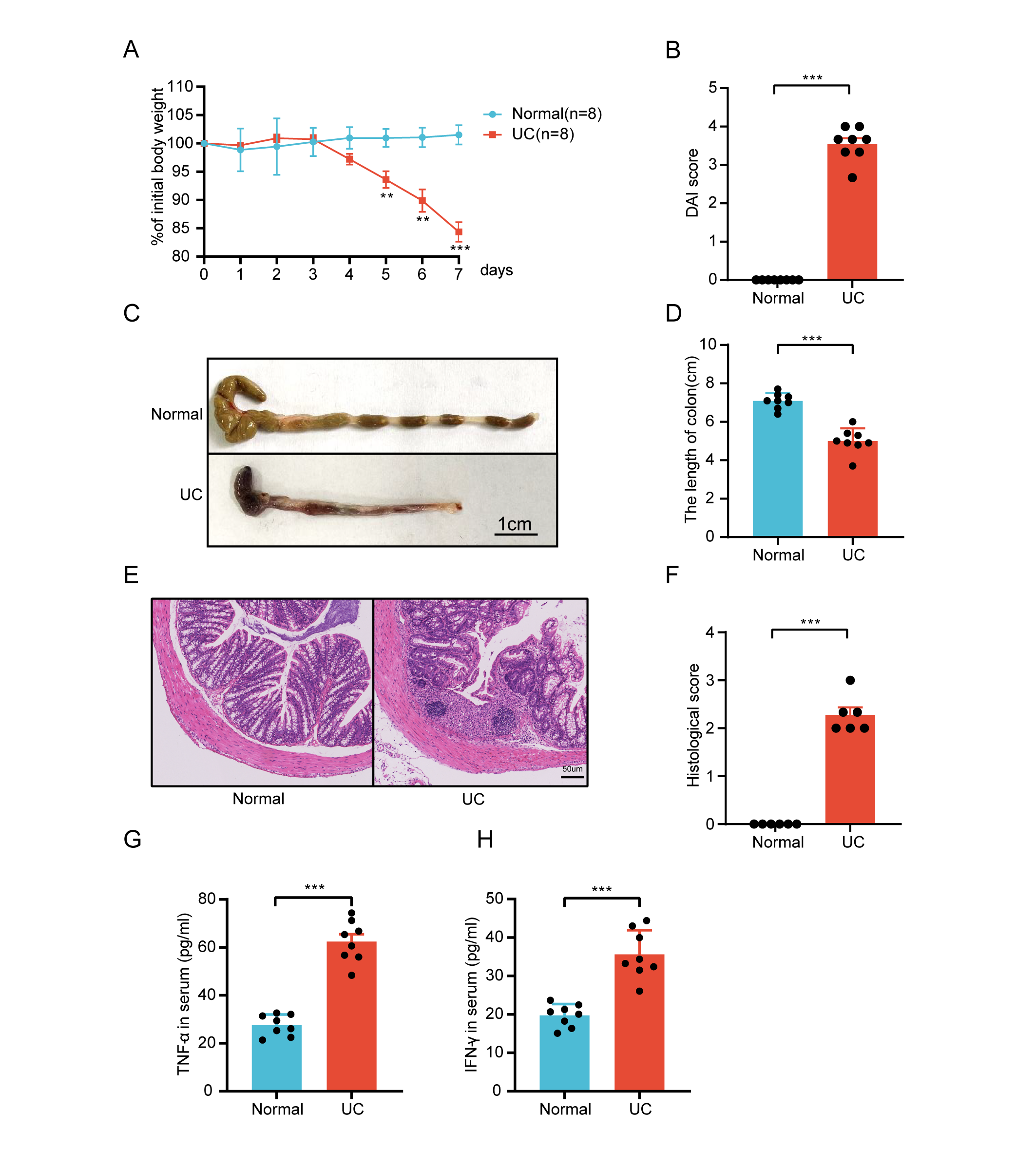

Supplement: Supplementary file 2 [file Image3.TIF]

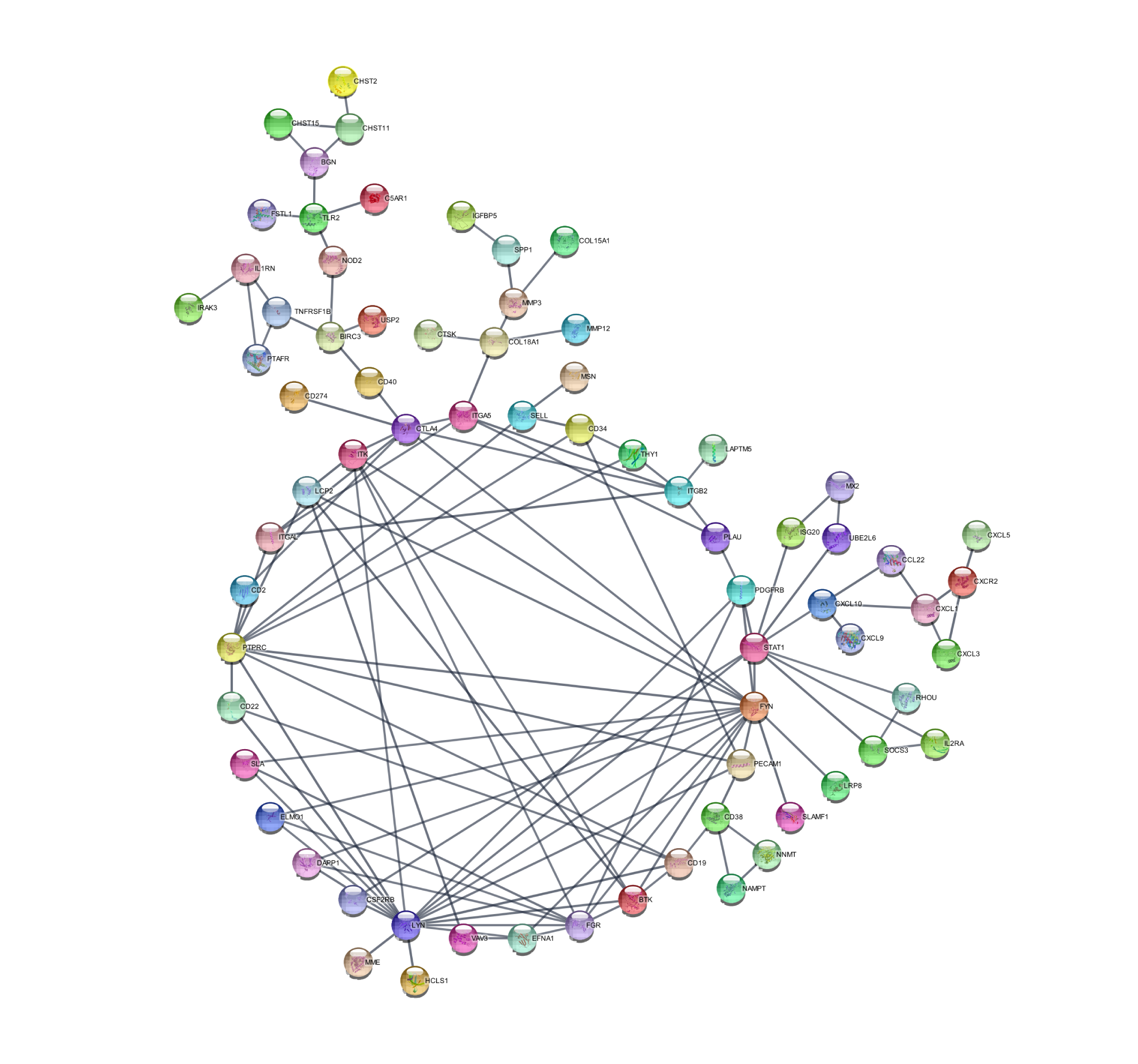

Supplement: Supplementary file 3 [file Image2.TIF]

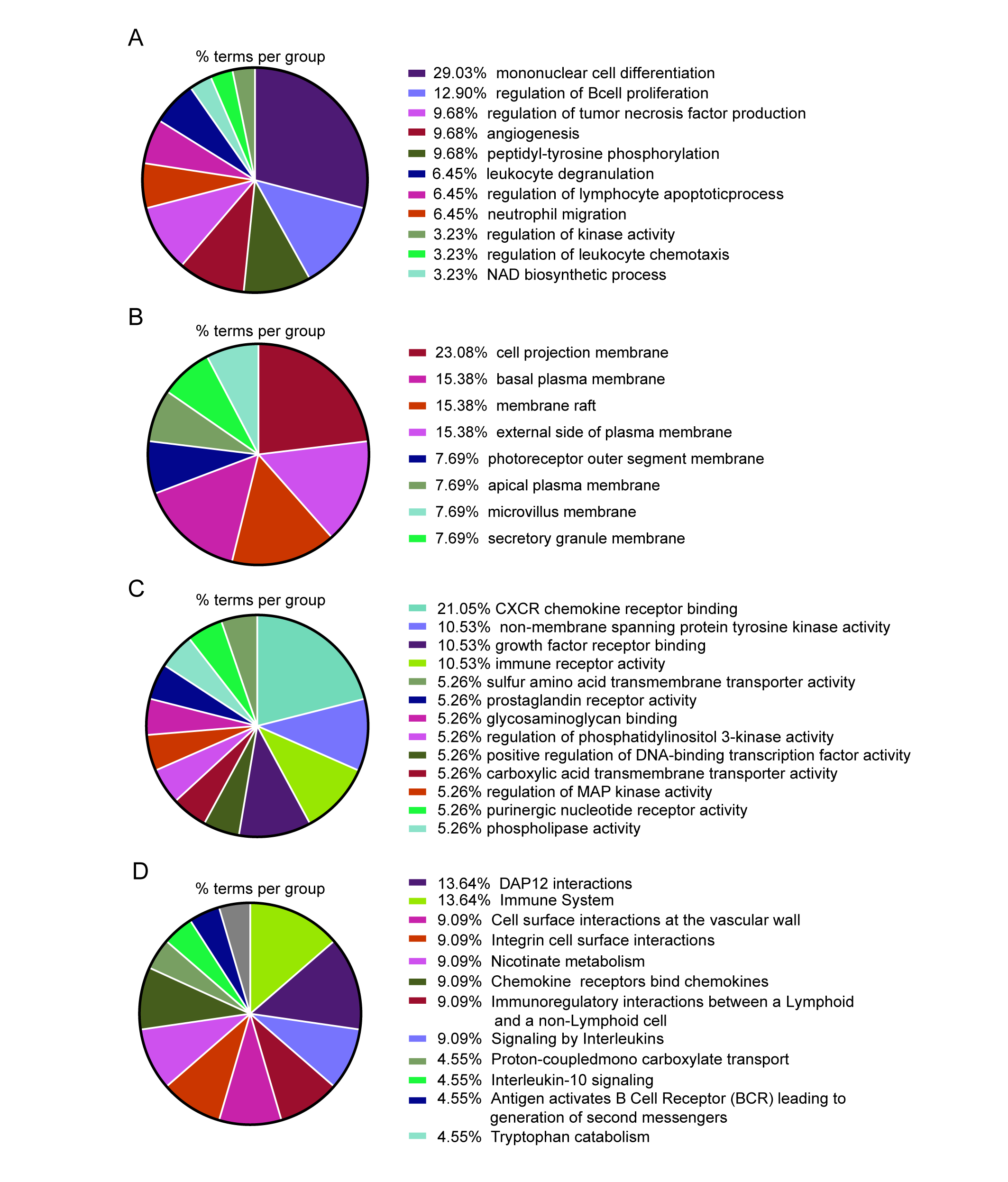

Supplement: Supplementary file 4 [file Image1.TIF]
