# Supplementary material for: Construction, bioinformatics analysis, and validation of competitive endogenous RNA networks in ulcerative colitis
Source: Front Genet. 2022 Aug 17;13:951243. doi: 10.3389/fgene.2022.951243 (PMC9428148; doi:10.3389/fgene.2022.951243)
Supplement: Supplementary file 5 [file Table2.DOCX]

Supplementary Table S2. Histological scores of colitis

| Feature | Score | Description |
| --- | --- | --- |
| Inflammation | 0 | None |
|  | 1 | Minimal multifocal inflammation (few foci) |
|  | 2 | Moderate multifocal inflammation (numerous foci) |
|  | 3 | Severe multifocal coalescing inflammation |
| Extent | 0 | None |
|  | 1 | Mucosa |
|  | 2 | Mucosa and submucosa |
|  | 3 | Transmural |
| Crypt damage | 0 | None |
|  | 1 | Basal 1/3 damaged |
|  | 2 | Basal 2/3 damaged |
|  | 3 | Only surface epithelium intact |
