# Supplementary material for: Construction, bioinformatics analysis, and validation of competitive endogenous RNA networks in ulcerative colitis
Source: Front Genet. 2022 Aug 17;13:951243. doi: 10.3389/fgene.2022.951243 (PMC9428148; doi:10.3389/fgene.2022.951243)
Supplement: Supplementary file 6 [file Table3.DOCX]

Supplementary Table S3.qRT-PCR primer sequences

| Gene Name | Primers (5`–3`) |
| --- | --- |
| *β-actin* | F: GGCTGTATTCCCCTCCATCG  R: CCAGTTGGTAACAATGCCATGT |
| *Ctla4* | F: GCTTCCTAGATTACCCCTTCTGC  R: CGGGCATGGTTCTGGATCA |
| *Itk* | F: GGAAGAAGCGCACGTTGAAG  R: ATGCACGACCTGAAAAGGGTA |
| *Fyn* | F: ACCTCCATCCCGAACTACAAC  R: CGCCACAAACAGTGTCACTC |
| *Itgb2* | F: CAGGAATGCACCAAGTACAAAGT  R: CCTGGTCCAGTGAAGTTCAGC |
| *Miat* | F: TTTGCCTTTCTGGTCTGTTCCTTCC  R: CCGCCATCATCCAAGCCGTTAG |
